# Genome-Wide Characterization of Nuclear Factor Y (NF-Y) Transcription Factors in Allohexaploid Oat

**DOI:** 10.3390/plants15162466

**Published:** 2026-08-14

**Authors:** Cailian Du, Yvkun Xue, Hao Wang, Qingbin Sun

**Affiliations:** 1 College of Life Sciences, Institute of Life Science and Green Development, Hebei University, Baoding 271000, China; ducailian26@163.com (C.D.); 15830923393@163.com (Y.X.); 2 Advanced Medical Research Institute, Shandong University, Jinan 250012, China

**Keywords:** allohexaploid oat, NF-Y transcription factor, genome-wide, abiotic stress

## Abstract

Nuclear factor Y (NF-Y) constitutes a pivotal transcription factor family that modulates plant growth and development as well as abiotic stress responses. Oat (*Avena sativa* L.) is an economically vital cereal crop and a major livestock forage globally. Nevertheless, the NF-Y gene family has not yet been systematically characterized in the oat genome. Here, we identified 36 *AsNF-Y* genes in the oat genome and categorized them into three distinct subfamilies (*NF-YA*, *NF-YB*, and *NF-YC*). Phylogeny, gene structure, duplication, collinearity, and conserved motif analyses revealed high evolutionary conservation of this gene family. Additionally, the identification of diverse *cis*-acting regulatory elements in the promoters of *AsNF-Y* genes, combined with their differential expression profiles under multiple abiotic stress conditions, indicated that *AsNF-Ys* serve as crucial regulators in modulating oat abiotic stress tolerance. Furthermore, preliminary functional validation via the TRV-VIGS system confirmed that two candidate genes, *AsNF-YC02* and *AsNF-YC06*, may positively regulate salt tolerance in oat. Collectively, our findings deepen the understanding of *NF-Y* genes in gramineous crops and supply promising candidates for the genetic improvement of oat stress tolerance and molecular breeding.

## 1. Introduction

The nuclear factor Y (NF-Y) transcription factor, also referred to as the CCAAT-binding factor (CBF) or heme activator protein (HAP), is ubiquitously distributed across eukaryotes. It assembles into a heterotrimeric complex consisting of three distinct subunits, namely NF-YA, NF-YB, and NF-YC [1,2]. While the NF-Y complex has been extensively characterized in animal systems, a striking distinction exists between plants and yeast/mammals: unlike the latter, where each NF-Y subunit is encoded by a single gene, plants harbor a large repertoire of NF-Y subunit-encoding genes [3]. This expanded genetic diversity facilitates the formation of a vast array of NF-Y complex isoforms, which endows plants with extensive regulatory plasticity and thereby greatly enhances their genetic diversity and environmental adaptability [4]. To date, members of the *NF-Y* gene family have been comprehensively identified across a broad spectrum of plant species, such as *Zea mays* [5], *Triticum aestivum* [6], *Sorghum bicolor* [7], *Glycine max* [8], and *Solanum lycopersicum* [9]. With respect to the regulatory mechanism of the NF-Y complex, a consensus has been reached that NF-YB and NF-YC subunits first assemble into a heterodimer in the cytoplasm. This dimer is then translocated into the nucleus, where it interacts with NF-YA to form a functional heterotrimeric complex. The resultant complex binds to the conserved CCAAT cis-elements within the promoters of target genes, thereby activating or repressing their transcription. Beyond this canonical pathway, distinct NF-Y subunits can also interact with other transcription factors to form non-canonical complexes, expanding the regulatory repertoire for the modulation of downstream gene expression [10].

Transcription factors play important roles in plant stress responses by integrating ABA, jasmonate, salicylic acid, ethylene, and ROS signaling [11]. Among them, NF-Y transcription factors act as pivotal regulators controlling diverse plant developmental processes (e.g., flowering and embryogenesis) and adaptive responses to abiotic stresses such as drought and salinity [2,8,12]. For instance, *AtNF-YB9*, the first plant *NF-Y* gene to be cloned, functions as a core regulatory TF that modulates seed development in *Arabidopsis thaliana*. Silencing of *ZmNF-YC12* in maize attenuated the activation of antioxidant systems, reduced soluble protein and proline accumulation, and elevated water loss rates, ultimately impairing drought tolerance and post-stress recovery capacity [5]. In soybean, *GmNF-YC14* assembles into a heterotrimer with *GmNF-YA16* and *GmNF-YB2* to activate the GmPYR1-mediated abscisic acid (ABA) signaling pathway, thereby enhancing stress tolerance [2]. Furthermore, upregulating *NF-Y* gene expression has evolved into a promising strategy for crop genetic improvement, with validated benefits reported across multiple crop species [13]. A prominent illustration is the overexpression of *NF-YC4*, which enhances protein accumulation and fortifies stress defense traits in soybean [14], rice [15], tobacco [16], and potato [17]. Collectively, these findings underscore the indispensable role of NF-Y-mediated transcriptional regulation in orchestrating diverse biological processes across major crop species.

Oat (*Avena sativa* L.), an allohexaploid species with the genome constitution AACCDD (2n = 6x = 42), belongs to the genus *Avena* of the Pooideae subfamily in the Poaceae family [18]. Endowed with superior agronomic traits, including cold tolerance, high nutritional value, strong stress resistance, and good palatability, oat is cultivated in approximately 42 countries worldwide [19]. To avoid competition with major cereal crops for prime arable land, oat germplasm is predominantly cultivated on marginal lands characterized by adverse growing conditions such as high salinity, drought, and extreme temperatures. Consequently, oat exhibits higher tolerance to abiotic stresses compared with staple crops like rice and wheat, rendering it a valuable genetic resource for mining abiotic stress-resistant genes in plants [20]. Fortunately, the release of a high-quality reference genome for oat has greatly accelerated functional genomic research in this species, providing a robust foundation for the exploration of abiotic stress resistance genes [18,21,22]. Accordingly, several important transcriptional factor families, such as WRKY [23], GRAS [24], NAC [25], TCP [26], and MADS [27], have been identified to be involved in abiotic stress processes in oats. However, the members of the NF-Y family associated with stress tolerance in oats are still largely unknown.

In this study, NF-Y transcription factors in oats were identified at the genome-wide level. A total of 36 *AsNF-Y* members were identified in allohexaploid oat, including 19 *AsNF-YAs*, 11 *AsNF-YBs*, and 6 *AsNF-YCs*. Systematic analyses were subsequently conducted to dissect their phylogenetic relationships, chromosomal location, gene structural features, conserved protein motifs, and intergenomic collinearity. Furthermore, we profiled the expression dynamics of *AsNF-Ys* in response to multiple abiotic stresses, including drought, heat, salinity, and alkalinity. Functional verification using the TRV-VIGS system revealed that *AsNF-YC02* and *AsNF-YC06* may positively regulate salt tolerance in oat. Collectively, these findings lay a solid foundation for subsequent functional validation of individual *AsNF-Y* genes and the construction of their molecular regulatory networks in mediating oat stress tolerance.

## 2. Results

### 2.1. Identification, Phylogenetic Analysis, and Chromosome Distribution of NF-Ys in Oat

#### 2.1.1. Identification and Phylogenetic Analysis of AsNF-Ys

**Figure 1:**
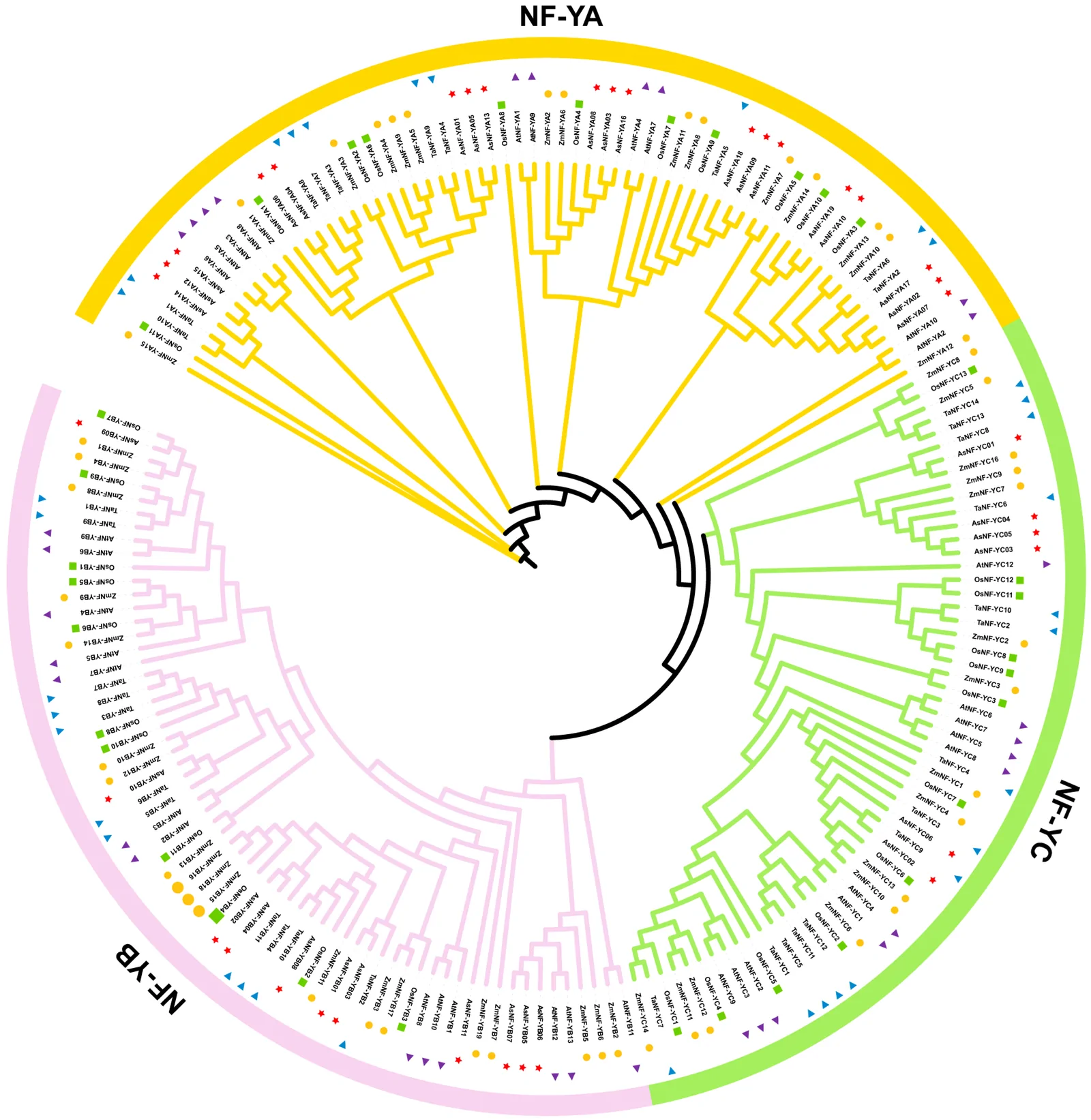
Phylogenetic classification of *NF-Ys* among oat, *Arabidopsis*, maize, wheat, and rice. Different colored arcs are used to distinguish different *NF-Y* subfamilies. Different shape symbols represent different species: green squares for rice, yellow circles for maize, red pentagrams for oat, and purple and blue triangles for Arabidopsis thaliana and wheat, respectively.

Combining BLASTP and HMMER searches, a total of 36 *AsNF-Y* genes with complete conserved domains were identified in the allohexaploid oat genome, including 19 *AsNF-YA*, 11 *AsNF-YB*, and 6 *AsNF-YC* members (Supplementary Tables S1 and S2). To comprehensively investigate the phylogenetic relationships and potential functional divergence of *NF-Y* family members, 33, 35, 34, and 49 *NF-Y* genes were retrieved from *Arabidopsis thaliana*, *Triticum aestivum*, *Oryza sativa*, and *Zea mays*, respectively (Supplementary Table S3). A phylogenetic tree was then constructed using MEGA 11.0 software (Supplementary Table S4). Phylogenetic analysis revealed that the 36 *AsNF-Ys* were clustered into three distinct clades, which exactly corresponded to the three *NF-Y* subfamilies (Figure 1). The number of members in each subfamily ranged from 6 (*NF-YC*) to 19 (*NF-YA*), suggesting differential expansion rates of the three NF-Y subfamilies during oat evolution.

#### 2.1.2. Basic Molecular Features of the AsNF-Y Family Members

The basic molecular features of the AsNF-Y family, including subfamily classification and chromosomal distribution, as well as the amino acid length, molecular weight (MW), and isoelectric point (pI) of encoded proteins, are summarized in Table 1. The amino acid lengths of AsNF-Y proteins span 127 to 477 residues. Their predicted pI values range from 4.50 to 10.92, among which 25 proteins (69.44%) have a pI above 7. These data reveal that most AsNF-Y proteins are basic proteins, and the AsNF-Y family possesses distinct structural and physicochemical diversity in allohexaploid oat.

### 2.2. Chromosome Distribution of AsNF-Ys

**Figure 2:**
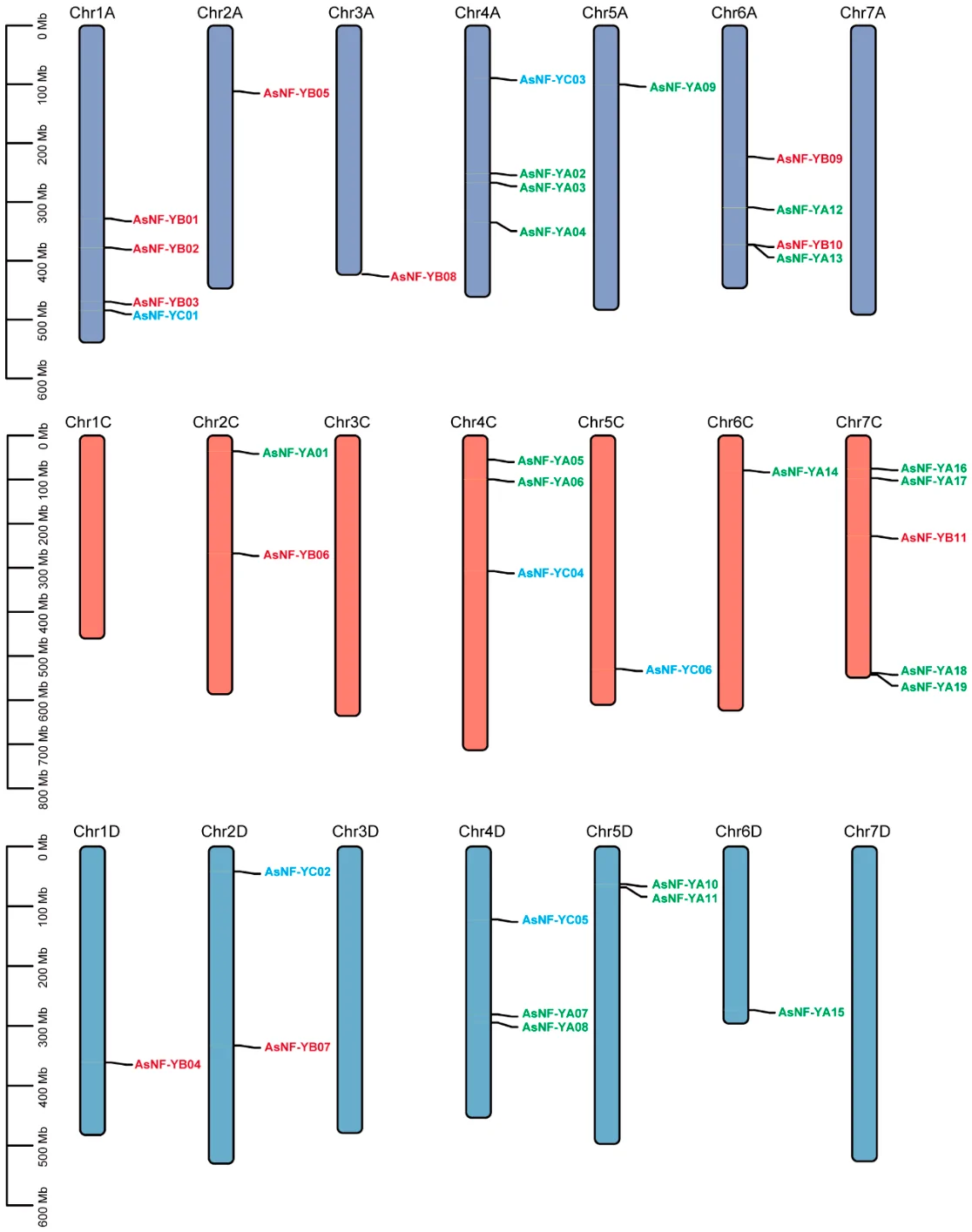
Chromosomal location of *AsNF*-Y genes.

Using the annotated allohexaploid oat genome, we characterized the chromosomal distribution of the 36 identified *AsNF-Y* genes (Figure 2). These genes exhibited uneven distribution across the 21 oat chromosomes. Chromosome 7C contained the highest number of *AsNF-Y* members (5 genes, ~13.89% of the total), followed by chromosomes 1A, 4A, and 6A, each harboring four genes (~11.11% of the total). By contrast, only a single *AsNF-Y* gene was detected on each of chromosomes 2A, 3A, 5A, 5C, 6C, 1D, and 6D. Further subgenomic distribution analysis indicated that the A, C, and D subgenomes harbor 15, 12, and 9 *AsNF-Y* genes, respectively.

### 2.3. Multiple Sequence Alignment of Three AsNF-Y Subfamilies

**Figure 3:**
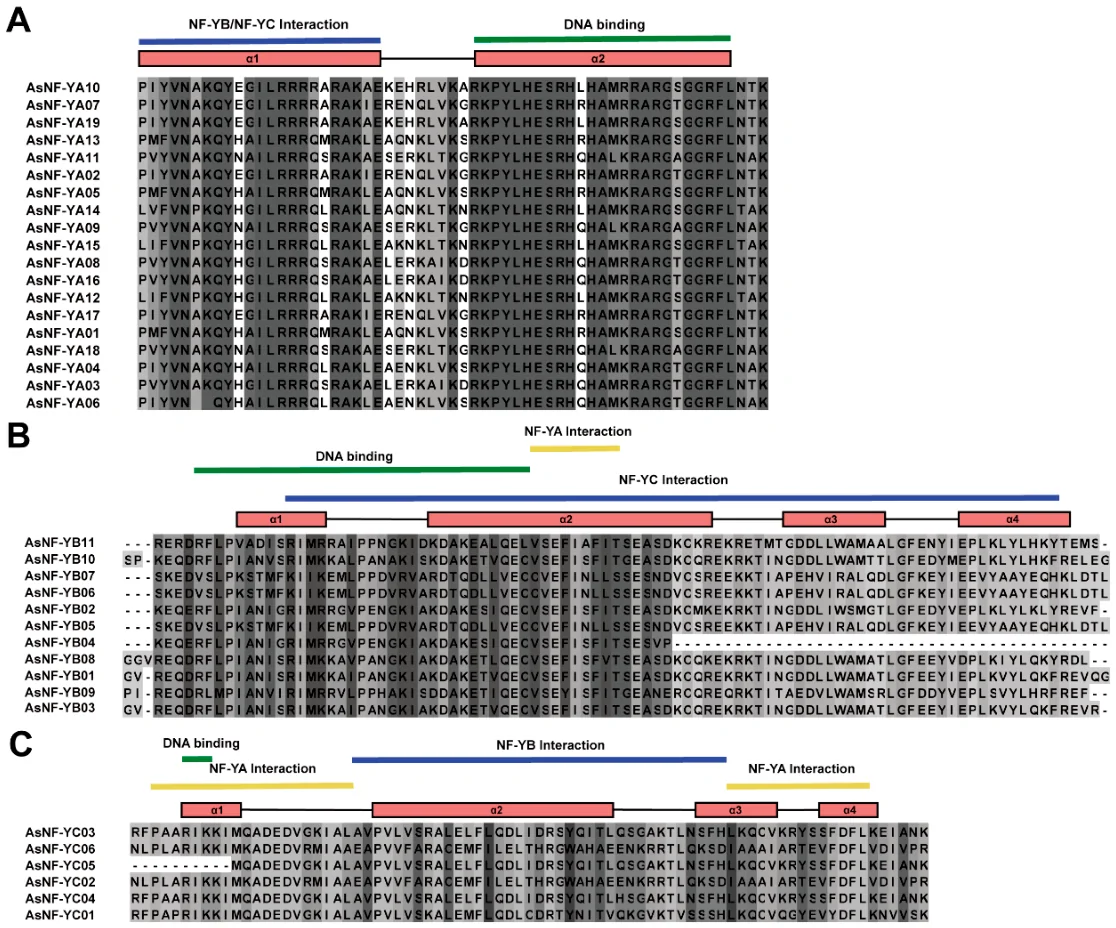
Multiple sequence alignments of AsNF-Y proteins. (**A**) The NF-YB/NF-YC interaction domain and DNA-binding domain of AsNF-YA subfamily; (**B**) The NF-YA/NF-YC interaction domain and DNA-binding domain of AsNF-YB subfamily; (**C**) The NF-YA/NF-YB interaction domain of AsNF-YB subfamily. These domains are indicated with different colored lines.

To characterize the conserved domains across distinct *AsNF-Y* subfamilies, multiple sequence alignment was performed using DNAMAN software (Version 10). The results revealed several conserved domains within AsNF-Y proteins, confirming their classification into the corresponding subfamilies (Figure 3). Specifically, AsNF-YA proteins harbored two conserved domains: one mediating protein–protein interactions with NF-YB/C subunits and the other serving as a DNA-binding domain (Figure 3A). Similarly, AsNF-YB proteins contained a DNA-binding domain and an additional domain responsible for interacting with NF-YA/C proteins (Figure 3B). As for AsNF-YC proteins, they also possessed a short DNA-binding domain and a domain that facilitates interactions with NF-YA/B; however, the conservation level of these domains was lower than that observed in AsNF-YA/B subfamilies, implying potentially more diverse functional roles in oat (Figure 3C). Collectively, the high homology of NF-Ys suggests that they may exert conserved biological functions. Meanwhile, this also indicates that genetic redundancy and functional differentiation might exist within this gene family.

### 2.4. Gene Structure and Conserved Motif Analyses of the AsNF-Y Gene Family

**Figure 4:**
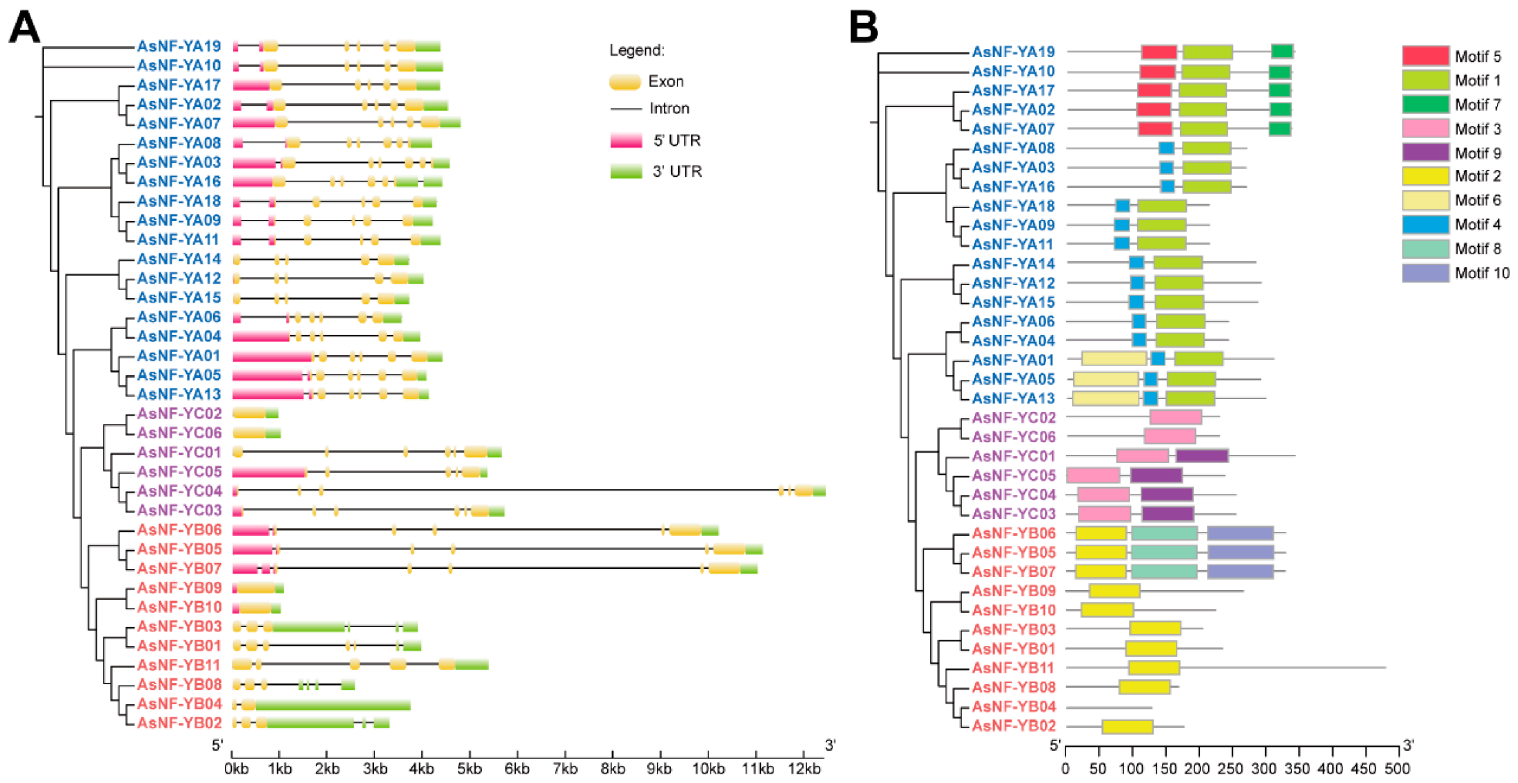
Phylogenetic relationships, gene structures, and conserved motifs of AsNF-Ys: (**A**) Phylogenetic relationship and gene structure of *AsNF-Ys*. Red and green boxes represent 5′ UTR and 3′ UTR, respectively. Yellow boxes represent exons, and black lines represent introns. (**B**) Phylogenetic relationship and conserved motifs of AsNF-Y proteins. The conserved motifs, numbers 1–10, are shown in different colored boxes. The details of each motif are presented in Supplementary Table S5.

To gain deeper insights into the structural characteristics and functional conservation of AsNF-Y proteins, we first conducted exon–intron structure analysis. A simplified phylogenetic tree was constructed, based on which the 36 AsNF-Y proteins were clustered into three groups. All identified *AsNF-Y* genes contained 1 to 6 introns (Figure 4A). Furthermore, genes clustered on the same branch of the phylogenetic tree exhibited similar exon–intron structures. Within each group, the location and length of introns showed minimal variation, whereas significant differences were observed between different groups. We further identified 10 conserved motifs in these proteins using the MEME motif search tool and analyzed their distribution patterns (Figure 4B, Supplementary Table S5). The results showed that the 10 most conserved motifs contained 15–58 amino acids. Among them, motif 6 had the fewest amino acids (15 amino acids). In addition, genes with high sequence similarity shared similar motif compositions. Such consistent motif arrangements may suggest potential functional conservation within the *AsNF-Y* gene family, though this hypothesis requires further experimental validation. Collectively, these findings reveal that the *AsNF-Y* gene family in oat exhibits high conservation at the level of gene structure and protein motif composition.

### 2.5. Gene Duplication and Collinearity Analysis of the AsNF-Y Genes

**Figure 5:**
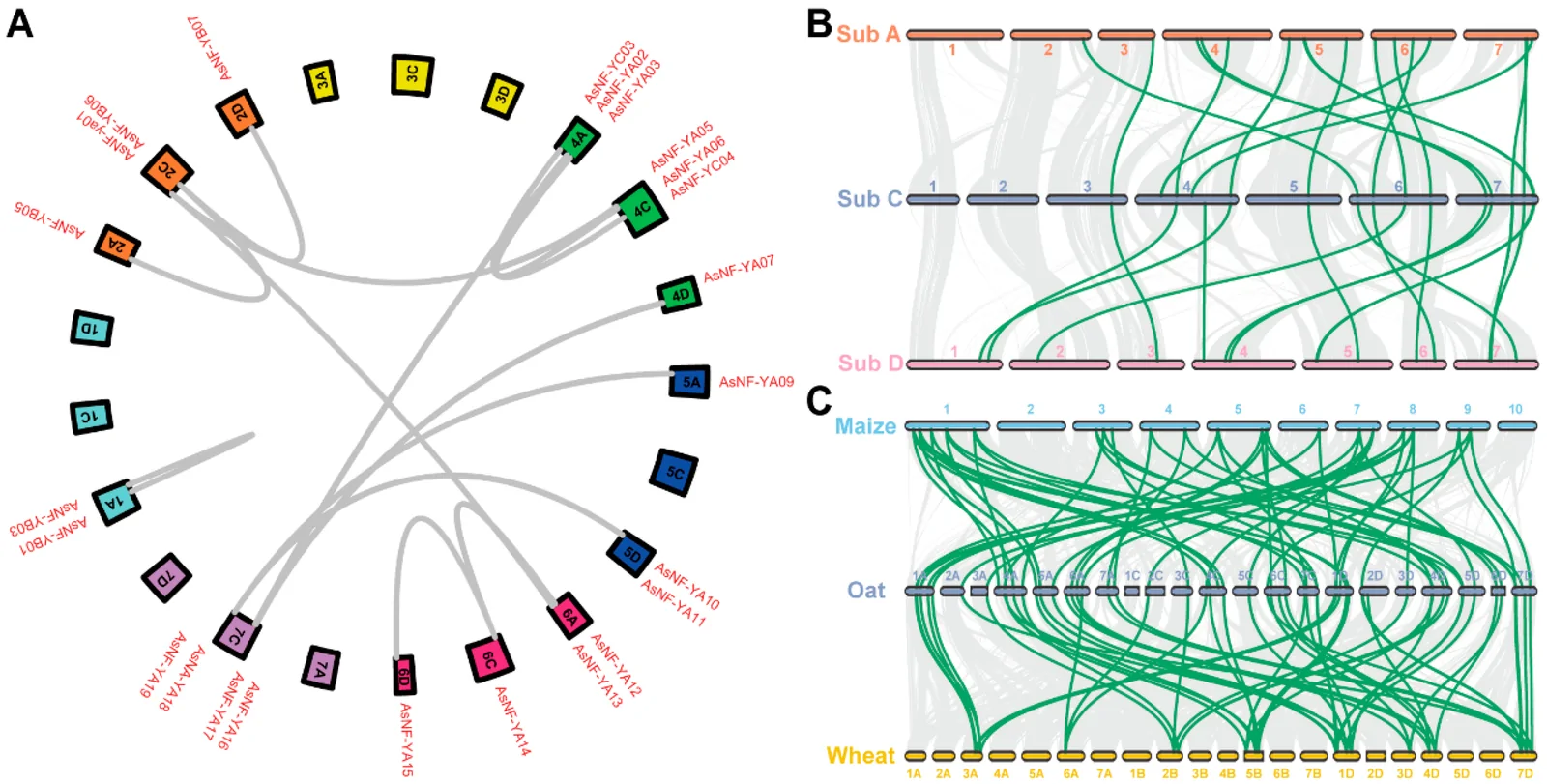
Gene duplication and collinearity analysis of the *AsNF-Ys*: (**A**) Chromosome localization of *AsNF-Y* duplicated genes in oat. The lines represent segmentally duplicated genes. (**B**) Synteny analyses of *AsNF-Y* genes among the subgenomes of oat. (**C**) Synteny analyses of the *NF-Y* genes among oat, wheat, and maize. The collinearity within oat and other species genomes is displayed by gray lines. The syntenic *NF-Y* gene pairs between oat and other species are highlighted with green lines. In panel (**B**), SubA, SubB and SubC represent the three subgenomes of allohexaploid oat, and the numbers 1–7 denote the chromosomes within each subgenome. In panel (**C**), the numbers 1–10, 1A–7A, 1B–7B, and 1C–7C denote the chromosomes of maize, oat and wheat, respectively.

Gene duplication provides raw materials and is the source of power for biological evolution. We systematically categorized duplication events of the 36 *AsNF-Y* genes using the ‘duplicate_gene_classifier’ program implemented in the MCScanX toolkit. As summarized in Supplementary Table S6, of the 36 *AsNF-Y* genes, 16 (44.4%) were assigned to dispersed duplications, another 16 (44.4%) to tandem duplications, and four (11.1%) to proximal duplications, whereas no WGD/segmental duplication events were detected. This classification aligns with the chromosomal distribution patterns (Figure 5A,B): four tightly clustered genes on chromosome 7C (*AsNF-YA16*/*AsNF-YA17*/*AsNF-YA18*/*AsNF-YA19*) were characterized as tandem duplicates. Likewise, three genes on chromosome 4A (*AsNF-YA02*/*AsNF-YA03*/*AsNF-YC03*) and three genes on chromosome 4C (*AsNF-YA05*/*AsNF-YA06*/*AsNF-YC04*) also arose from tandem duplication events. As listed in Supplementary Table S7, the Ka/Ks ratios of all examined duplicated AsNF-Y gene pairs were substantially lower than 1, varying from 0.14 to 0.64. This finding reveals that the AsNF-Y gene family has evolved mainly under strong purifying selection, consistent with the conserved gene structures and motif profiles presented in Figure 4.

To further elucidate the evolutionary trajectory of the *AsNF-Y* gene family across cereal crops, we performed interspecific collinearity analysis among oat, maize, and wheat. The results demonstrated extensive collinear gene blocks among the three species. Specifically, a genome-wide collinearity analysis detected a total of 29,720 syntenic gene pairs between oat and maize, accounting for 45.70% of the total gene number in oat. Additionally, 32,207 syntenic gene pairs were identified between oat and wheat, representing 49.53% of oat’s total genes (Figure 5C). Notably, *AsNF-Y* genes were found to be associated with at least two or more syntenic pairs, particularly between wheat and oat. For instance, 32 *AsNF-Y* genes (88.89%) formed multiple homologous pairs with their wheat orthologs, and 29 genes (80.56%) exhibited this pattern with maize orthologs (Figure 5C), indicating that these *AsNF-Y* genes may have played critical roles in evolutionary processes and were preferentially retained during successive rounds of gene duplication events.

### 2.6. Cis-Elements in the Promoter of the AsNF-Y Genes

**Figure 6:**
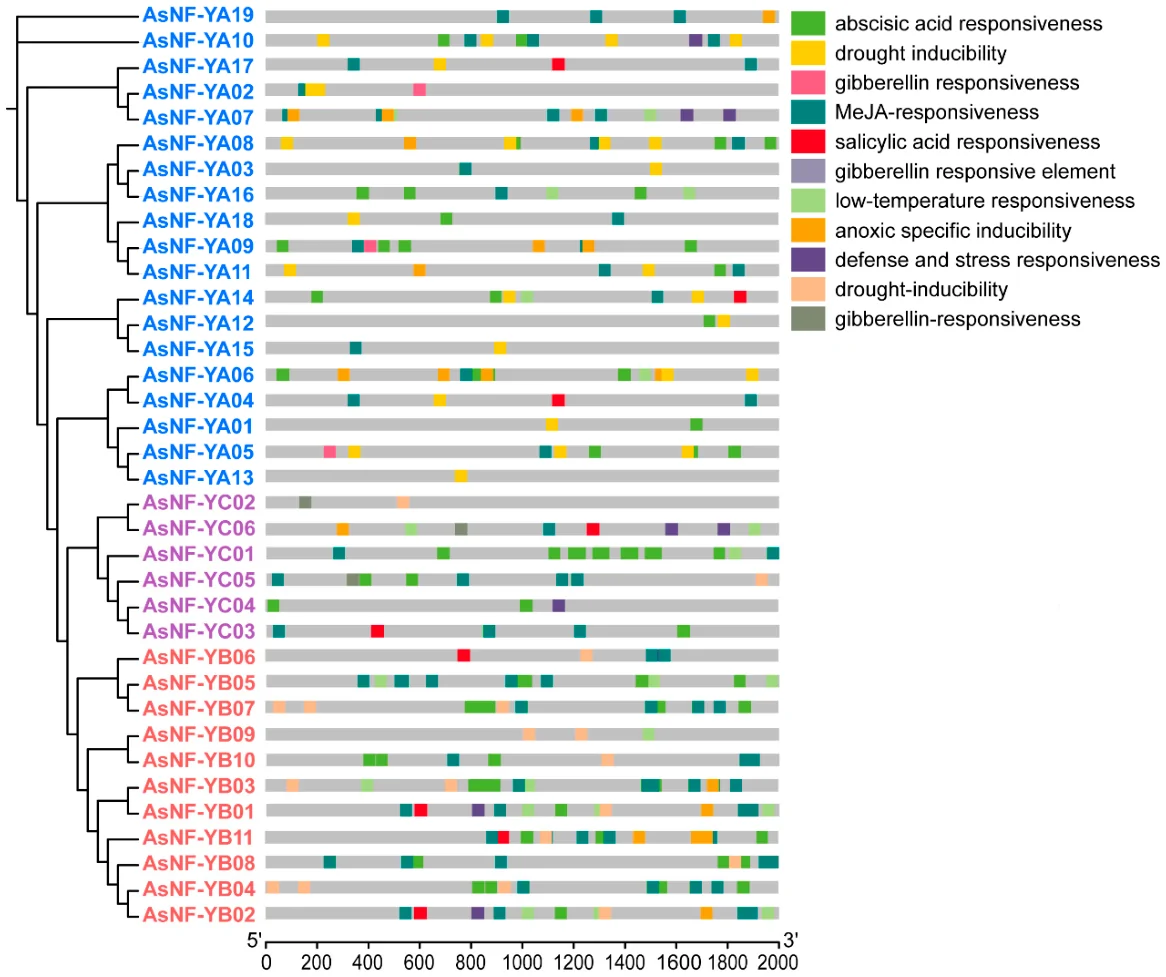
Phylogenetic relationships and *cis*-acting elements in promoters of *AsNF-Y* genes. Detailed information on the *cis*-acting elements contained in each *AsNF-Y* gene is provided in Supplementary Table S2.

To elucidate the potential transcriptional regulatory mechanisms governing *AsNF-Y* genes, we identified the *cis*-acting elements in their promoter regions using the PlantCare database (Supplementary Table S8), and the results revealed a total of 16 major categories of *cis*-acting regulatory elements across the *AsNF-Y* gene family. These elements were primarily associated with plant growth and development, hormone responsiveness, and stress tolerance. Specifically, the identified *cis*-elements included those involved in abscisic acid responsiveness (ABRE), drought stress responsiveness (DRE/CRT), methyl jasmonate (MeJA) responsiveness, salicylic acid responsiveness, gibberellin responsiveness (TATC-box), low-temperature responsiveness (LRE), anoxic-specific inducibility, and defense and stress responsiveness (Figure 6). Among them, light-responsive cis-regulatory elements were the most abundant and classified as the largest category. Hormone-related elements (e.g., MeJA- and ABA-responsive elements) constituted the second most prevalent category. Nevertheless, subtle yet distinct differences were observed among individual gene members. For instance, the drought stress-responsive element was detected in 19 *AsNF-Y* genes, whereas the gibberellin-responsive element was present in 23 members (Supplementary Table S9). Collectively, these results indicate that *AsNF-Y* genes may be regulated by multiple hormones and stress signals, potentially improving oat stress tolerance.

### 2.7. Expression Profiling of AsNF-Y Genes Under Various Abiotic Stress

**Figure 7:**
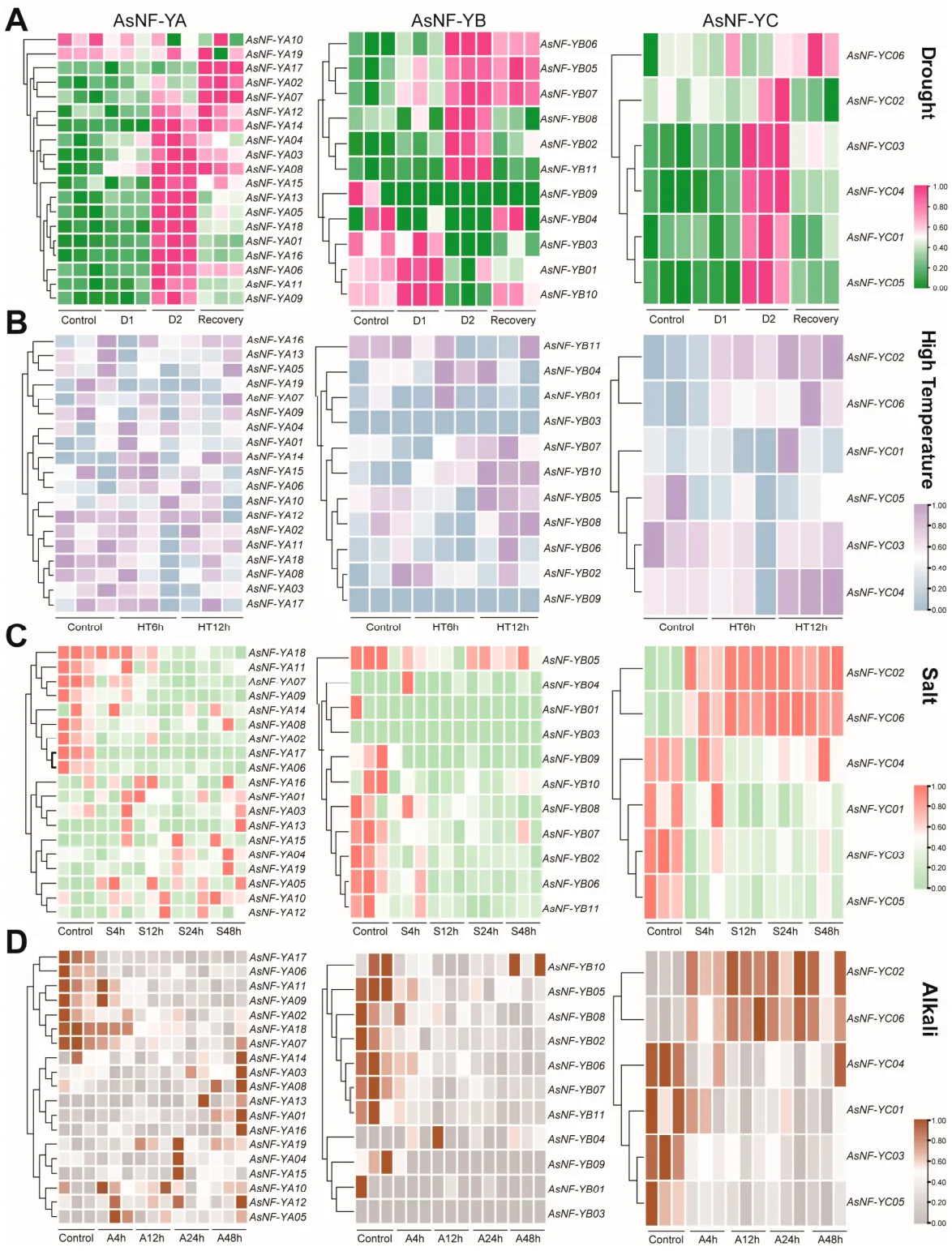
Expression patterns of *AsNF-Y* genes under different abiotic stresses: (**A**–**D**) Gene expression analysis of *AsNF-Y* genes under drought (**A**), high temperature (**B**), salt (**C**), and alkali (**D**) stress. The transcriptomic data in Panel (**A**) were obtained from a previous study. D1 and D2 represent mild drought and severe drought, respectively, and Recovery denotes rewatering [28]. The transcriptomic data in Panels (**C**,**D**) were also derived from a previous study [29]. S and A represent salt stress and alkali stress, respectively, and the numbers indicate the time into stress treatment. The transcriptomic data in Panel (**B**) were newly generated in this study, and the raw data have been deposited in the CNCB database (PRJCA070426). HT6h and HT12h represent 6 h and 12 h of high-temperature treatment (38 °C), respectively.

To investigate the response of *AsNF-Y* genes to abiotic stresses, we further analyzed the expression patterns of *AsNF-Y* genes under four different abiotic stress conditions, including drought, high temperature, salt, and alkali stress. The results demonstrated that, after excluding unexpressed genes with FPKM values < 0.5, the expression levels of *AsNF-Y* genes varied significantly under the four stress conditions (Figure 7, Supplementary Tables S10–S13). Specifically, the expression of most *AsNF-YA/C* genes was significantly upregulated under drought stress, with a more sensitive response to severe drought stress, such as *AsNF-YA16* and *AsNF-YC04* (Figure 7A). Under high-temperature stress, the expression of *AsNF-YB11* was significantly inhibited, while that of *AsNF-YC02* was induced, implying that these two genes may exert regulatory roles in the adaptation of oats to high-temperature stress (Figure 7B). Notably, the expression trends of all *AsNF-Y* genes were nearly identical under salt and alkali stress. Meanwhile, the expression of most *AsNF-YB* genes exhibited a marked downward trend under saline–alkali stress. In contrast, the expression of *AsNF-YC02* and *AsNF-YC06* was rapidly upregulated in response to saline–alkali stress, suggesting that these two genes may play pivotal roles in mediating the tolerance of oats to saline–alkali stress (Figure 7C,D). Taken together, these results demonstrate that *AsNF-Y* genes exhibit distinct and divergent expression patterns in response to different abiotic stresses in oats, implying their critical regulatory roles in mediating oat abiotic stress tolerance.

### 2.8. AsNF-YC02 and AsNF-YC06 May Positively Modulate Salt Tolerance of Oat

**Figure 8:**
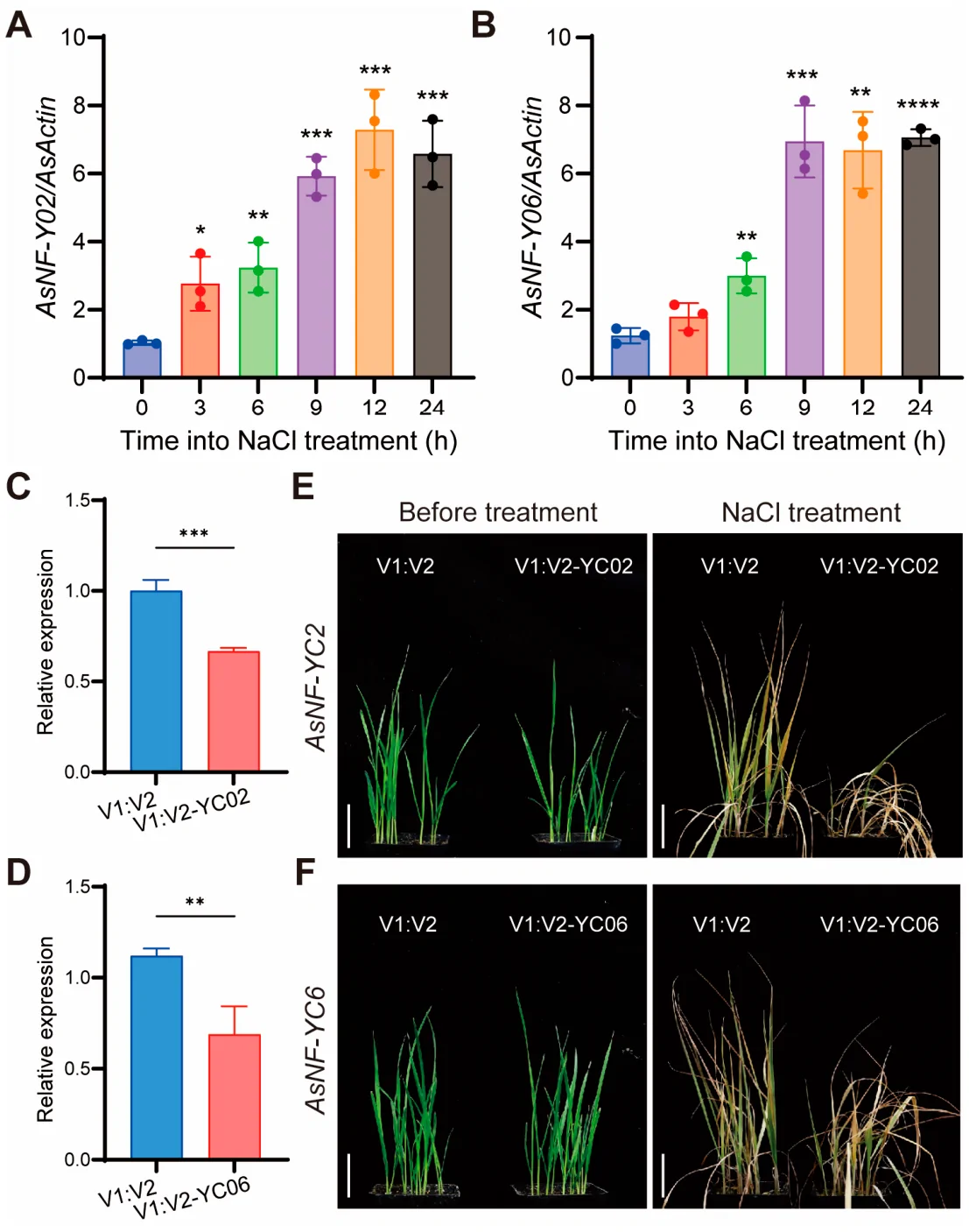
Preliminary functional validation of *AsNF-YC02* and *AsNF-YC06*: (**A**,**B**) Expression patterns of *AsNF-YC02* and *AsNF-YC06* under salt stress detected by RT-qPCR. Values are means ± SD from three independent experiments. The asterisks indicate significance, as determined by a one-way ANOVA analysis (* *p* < 0.05; ** *p* < 0.01; *** *p* < 0.001; **** *p* < 0.0001). (**C**,**D**) Expression levels of *AsNF-YC02* and *AsNF-YC06* in control (V1:V2) and TRV-VIGS-silenced plants (V1:V2-YC02, V1:V2-YC06). The data for each bar represent gene expression levels from six independent seedlings. The asterisks indicate significance, as determined by Student’s *t*-test (** *p* < 0.01; *** *p* < 0.001). (**E**,**F**) Phenotypes of control (V1:V2) and TRV-VIGS-silenced plants (V1:V2-YC02, V1:V2-YC06) before and after salt treatment. Two independent biological replicates were performed, and representative photographs were presented. Bar = 5 cm.

As shown in Figure 7, the transcript levels of *AsNF-YC02* and *AsNF-YC06* were significantly upregulated under saline–alkali stress, suggesting that these two genes play vital roles in the stress response of oat. To further verify their expression characteristics under salt stress, we performed RT-qPCR analysis. The results revealed that the transcript abundance of both genes increased dramatically after salt treatment, which was highly consistent with the transcriptome profiling data (Figure 8A,B).

Subsequently, we constructed gene knockdown lines of *AsNF-YC02* and *AsNF-YC06* via the tobacco rattle virus-mediated virus-induced gene silencing (TRV-VIGS) system (Figure 8C,D, Supplementary Table S14). Under normal growth conditions, no visible phenotypic differences were detected between control plants and the VIGS knockdown lines. However, when subjected to salt stress, the knockdown lines displayed obviously weakened salt tolerance (Figure 8E,F). Compared with the control group (V1:V2), the survival rate, dry weight, and plant height were significantly reduced in the VIGS-knockdown group (V1:V2-C02 or V1:V2-C06). We further determined the contents of Na^+^ and K^+^. The Na^+^ concentration was significantly higher, whereas the K^+^ concentration was markedly lower in the VIGS-knockdown group relative to the control, resulting in a higher Na^+^/K^+^ ratio (Supplementary Figure S1). Collectively, these findings preliminarily demonstrate that *AsNF-YC02* and *AsNF-YC06* may act as positive regulators of salt stress tolerance in oat, possibly by maintaining intracellular ion homeostasis.

## 3. Discussion

The NF-Y transcription factors play important roles in the regulation of plant growth, development, and stress responses. To date, the *NF-Y* gene family has been comprehensively characterized at the genome-wide level in multiple plant species; however, a systematic analysis of the evolutionary relationships among its members remains lacking in the allohexaploid oat. In this study, we identified all members of the *AsNF-Y* gene family and elucidated their evolutionary relationships at the genome-wide scale in oat. Expression profiling revealed that most *AsNF-Y* genes were responsive to diverse abiotic stresses, and two candidate genes, *AsNF-YC02* and *AsNF-YC06*, may positively regulate salt tolerance in oat. Collectively, these findings provide a valuable framework for further dissecting the biological functions of *AsNF-Y* family members.

Oat serves as both an economically valuable cereal and a key forage for livestock worldwide. Notably, it exhibits robust stress tolerance (e.g., drought, cold, and saline–alkali tolerance) and can be cultivated across diverse soil types and marginal regions with adverse growing conditions [19,30]. The release of a high-quality reference genome for oat has greatly accelerated functional genomic research in this species, providing a robust foundation for the exploration of abiotic stress resistance genes [18,21,22]. For instance, the AsDOF25-AsHSFA2c-AsAGO1 module plays a pivotal role in regulating the balance between oat growth and development and drought stress response [28]. In this study, expression profiling under four abiotic stresses (drought, high temperature, salt, and alkali) revealed distinct and divergent expression patterns of *AsNF-Y* genes, highlighting their specific roles in stress response regulation. For example, under drought stress, most *AsNF-YA/C* genes (e.g., *AsNF-YA16*, *AsNF-YC4*) were significantly upregulated, especially under severe drought conditions. This is consistent with previous findings in maize, where *ZmNF-YC12* positively regulates drought tolerance by enhancing antioxidant systems and proline accumulation [5]. Drought stress leads to substantial yield reductions in agricultural systems annually; thus, the identification of drought-associated regulatory genes is of great importance [31,32]. It is speculated that *AsNF-YA16* and *AsNF-YC4* may serve as positive regulators of drought tolerance in oat, which deserves further functional validation.

In this study, we found that *AsNF-YC02* and *AsNF-YC06* may positively regulate salt tolerance in oat, possibly by maintaining intracellular ion homeostasis (Figure 8). A prior study reported that the AsZAT18–AsWRKY49–AsSOS2 module also enhances oat salt tolerance through the regulation of ion homeostasis [29]. Presumably, the superior salt tolerance of oat relative to staple cereals, including wheat, rice, and maize, may be largely attributable to its capability to sustain ion homeostasis under salt stress. Indeed, maintenance of ion homeostasis constitutes one of the core adaptive strategies employed by plants to cope with salt stress. For example, *ZmHAK17*, which encodes a Na^+^-selective transporter, facilitates maize seed germination under saline conditions by sustaining ion homeostasis [33]. Accordingly, elucidating the mechanisms through which AsNF-YC02 and AsNF-YC06 modulate ion homeostasis under salt stress may represent a promising direction for future research.

Despite the multi-layered systematic characterization of the *AsNF-Y* gene family at the genomic, transcriptomic, and preliminary functional levels, several unresolved questions and limitations remain in this work. First, functional validation in the present work was limited to the salt tolerance of two *AsNF-YC* genes using the TRV-VIGS system. Second, the protein interaction networks among distinct AsNF-Y subunits and their downstream target genes remain uncharacterized. Third, as an allohexaploid crop with three subgenomes, oat harbors a large number of homologous *AsNF-Y* copies generated by polyploidization, and the subfunctionalization or neofunctionalization differentiation of these paralogous genes has not been clarified in this study. Therefore, future investigations may exploit the promising stress-tolerant *AsNF-Y* candidate genes identified herein to develop functional molecular markers for marker-assisted selection breeding. Introgression of favorable *AsNF-Y* alleles into elite oat cultivars holds the potential to generate novel varieties with enhanced tolerance to drought and saline–alkali stress.

## 4. Materials and Methods

### 4.1. Identification of AsNF-Y Family Members in Oat

The oat genome version used in this study is OT3098_v2, which was retrieved from the Oatomics database (http://www.oatomics.com/germplasm_info, accessed on 1 December 2025). According to the updated classification criteria for *NF-Y* transcription factors in *Arabidopsis thaliana* [34], amino acid sequences were retrieved from TAIR (http://www.arabidopsis.org, accessed on 1 December 2025). An initial search for *NF-Y* transcription factors in oat was conducted by using the protein sequences of *A. thaliana NF-Ys* as queries to BLAST against the oat protein database downloaded from http://www.oatomics.com/Download (accessed on 1 December 2025), employing the local BLAST software (version 2.2.24). Hits with a significant E-value ≤ 10−10 were extracted from the oat database via a custom Perl script. Subsequently, the protein sequences were analyzed using the online SMART tool (http://smart.embl-heidelberg.de, accessed on 1 December 2025) to verify the presence of conserved domains specific to the NF-Y family. The conserved domain sequences of NF-YA (PF02045) and NF-YB/NF-YC (PF00808) were retrieved from the Pfam database (http://pfam-legacy.xfam.org, accessed on 1 December 2025). The resulting sequences were designated as candidate *AsNF-Y* transcription factors in oat, and the identified *AsNF-Ys* were named based on their top-to-bottom order within the scaffolds.

### 4.2. Alignments, Phylogenies, Gene Structures, and Motif Analysis of AsNF-Ys

Sequences were aligned using MAFFT, and ambiguous aligned sites were trimmed with trimAl [35]. The maximum likelihood (ML) phylogenetic tree was constructed by IQ-TREE2 [36]. The best-fit amino acid substitution model was automatically selected by ModelFinder. Branch support was assessed using ultrafast bootstrap with 1000 replicates. The gene structures of *AsNF-Y* members from oat were generated by comparing the full-length coding sequences (CDSs) with their corresponding genomic DNA sequences using the Gene Structure Display Server (GSDS, http://gsds.cbi.pku.edu.cn, accessed on 1 January 2026) [37]. Additionally, the conserved motifs of all *NF-Y* members were analyzed using the online MEME tool (http://meme.nbcr.net/meme/tools/meme, accessed on 1 January 2026) with the following parameter settings: optimal motif width of 10–200 amino acids, any number of motif repetitions, and a maximum of 10 motifs to be identified.

### 4.3. Chromosomal Distribution of AsNF-Y Genes in Oat

The chromosome location of each *AsNF-Y* gene was retrieved from the GFF3 file of the oat genome (http://www.oatomics.com/Download, accessed on 1 December 2025) and then visualized on oat chromosomes using TBtools [38].

### 4.4. Gene Duplication and Synteny Analysis

Homologous gene pairs and syntenic relationships within the *AsNF-Y* gene family in oat were identified using the Multiple Collinearity Scan toolkit (MCScan) software (Version X) with default parameters. The syntenic relationships of *AsNF-Y* genes between oat and two other cereal crops (wheat and maize) were further explored via MCScanX with default settings. To classify the types of gene duplication events, the ‘duplicate_gene_classifier’ program in the MCScanX package was employed with default parameters. The program categorizes gene pairs into five types: singleton (Code 0), dispersed (Code 1), proximal (Code 2), tandem (Code 3), and WGD/segmental (Code 4), based on their chromosomal positions and syntenic relationships. To estimate the selection pressure acting on duplicated *AsNF-Y* gene pairs, the ratio of non-synonymous (Ka) to synonymous (Ks) substitution rates was calculated using the ‘Simple Ka/Ks Calculator’ program implemented in the TBtools software suite (v2.472). Additionally, the gene duplication events of *AsNF-Y* in oat, as well as the homologous genetic relationships of *AsNF-Y* genes among oat, wheat, and maize, were visualized using the advanced Circos plot function in TBtools software. These syntenic gene pairs are restricted to *NF-Y* family members rather than genome-wide collinear blocks.

### 4.5. Analysis of the AsNF-Y Gene Promoter in Oat

A 2000 bp nucleotide sequence upstream of the start codon of each *AsNF-Y* gene was extracted from the oat genome sequence and submitted to the PlantCARE database (https://bioinformatics.psb.ugent.be/webtools/plantcare/html, accessed on 1 February 2026) for cis-regulatory element prediction. The predicted cis-regulatory elements were classified based on their regulatory functions, and those associated with hormones and environmental stresses were visualized using TBtools software.

### 4.6. Transcriptome Sequencing Data Analysis

Transcriptome data under high-temperature stress were sequenced by Shanghai OE Biotech Co., Ltd. (Shanghai, China) and have been deposited in the CNCB database (PRJCA070426). Datasets for other stress treatments (drought, salt, and alkali) were retrieved from published studies [28,29]. Sequence reads were trimmed using Fastp (v0.20.1). Clean RNA-seq reads from samples were mapped to the “Oat_OT3098_v2” (http://www.oatomics.com/home) reference genome using HISAT2 (v2.2.1). Gene expression levels were quantified with StringTie (v2.1.6) based on fragments per kilobase of exon per million mapped fragments (FPKM) values, using the parameters ‘-G-e-A’. Differentially expressed genes (DEGs) were identified using the DESeq2 R package (v1.38.3) with default parameters, then further filtered with a minimum twofold change (|log2FoldChange| ≥ 1) and FPKM > 0.5. DESeq2 is designed to work with raw read counts rather than FPKM values. Heatmaps were generated using log (FPKM + 1)-transformed values.

### 4.7. Reverse Transcription Quantitative PCR (RT-qPCR) Analysis

Total RNA was extracted from plant tissues using Trizol reagent (CWBIO, Beijing, China) following the manufacturer’s instructions strictly. First-strand cDNA synthesis was performed with a reverse transcription kit (TIANGEN Biotech, Beijing, China) using the extracted total RNA as the template. Quantitative real-time PCR (qPCR) was conducted on a LightCycler (Roche, IN, USA) using SYBR Green I dye-based qPCR enzyme (TIANGEN Biotech, Beijing, China). Primers for target genes and reference genes were designed and synthesized by Sangon Biotech (Shanghai, China). All primers used in qPCR are listed in Supplementary Table S15. The relative expression levels of target genes were calculated using the 2^−ΔΔCt^ method.

### 4.8. Virus-Induced Gene Silencing (VIGS) Assay

The VIGS assays were conducted following previously described protocols [28,29,39]. Briefly, two fragments located within the open reading frames (ORFs) of target genes (*AsNF-YC02* and *AsNF-YC06*) were amplified by PCR and subcloned into the TRV2 vector using the NC cloning kit (NCBiotech). For infiltration, *Agrobacterium tumefaciens* strains harboring *pTRV1* and different *pTRV2*-derived vectors (empty *TRV2*, *V2-YC02*, and *V2-YC06*) were mixed at a 1:1 ratio in infiltration medium supplemented with 19.62 mg L^−1^ acetosyringone (AS) (Coolaber, Beijing, China), 400 mg L^−1^ cysteine (Cys) (Coolaber, Beijing, China), and 5 mL L^−1^ Tween-20 (Sangon, Shanghai, China). Germinating oat seeds were vacuum-infiltrated with the bacterial mixture at 20 kPa for 5 min, followed by overnight co-cultivation at 28 °C with shaking at 180 rpm. After co-cultivation, seeds were rinsed with sterile water to remove surface-attached agrobacteria and subsequently sown in soil. All primer sequences are provided in Supplementary Table S15.

### 4.9. Plant Growth Conditions and Stress Treatment

For the TRV-VIGS assay, the oat cv. ‘Bayou 18’ was used in this study. The infected seeds were planted in mixed soil (vermiculite–nutrient soil = 1:1) and cultured in an incubator at 25 °C/18 °C (day/night), with a 16 h/8 h light/dark photoperiod and 50% relative humidity. Seedlings were irrigated with 200 mM salt solution (NaCl:Na_2_SO_4_ molar ratio = 5:1) at the three-leaf and one-heart stage. Photographs were taken, and relevant growth phenotypes were recorded 8 days post-treatment. For transcriptome sampling, seedlings of oat cv. ‘Pinyan 6’ at the three-leaf and one-heart stage were subjected to high-temperature stress (38 °C) for 6 h and 12 h, and leaves were harvested for transcriptome sequencing.

### 4.10. Measurement of Ion Content (Na^+^ and K^+^) 

Samples collected for ion content measurement were processed as previously described [29,40], and a flame photometer (FP6410, INESA) was used to measure the Na^+^ and K^+^ content.

## Figures and Tables

**Table 1:** Basic characteristics of the AsNF-Ys.

Gene ID	Gene Name	Protein Length (aa)	CDS Length (bp)	Molecular Weight (Da)	PI	Location of Start	Location of End
2Cg0000310	*AsNF-YA01*	309	930	33.92	10.2	35,914,874	35,919,264
4Ag0001043	*AsNF-YA02*	335	1008	35.95	9.65	251,414,656	251,419,151
4Ag0001216	*AsNF-YA03*	267	804	28.87	9.56	267,345,751	267,350,286
4Ag0002255	*AsNF-YA04*	242	729	26.43	10.13	334,872,628	334,876,550
4Cg0000757	*AsNF-YA05*	289	870	31.6	10.07	54,892,658	54,896,722
4Cg0001284	*AsNF-YA06*	242	729	26.38	10.11	99,762,993	99,766,524
4Dg0001404	*AsNF-YA07*	335	1008	35.96	9.68	280,647,618	280,652,390
4Dg0001569	*AsNF-YA08*	269	810	29.01	9.17	294,394,757	294,398,918
5Ag0001006	*AsNF-YA09*	213	642	23.05	8.87	100,405,630	100,409,815
5Dg0000553	*AsNF-YA10*	336	1011	36.47	9.21	63,116,399	63,120,793
5Dg0000593	*AsNF-YA11*	213	642	23.1	8.63	68,338,765	68,343,124
6Ag0001782	*AsNF-YA12*	290	873	31.27	10.83	309,231,547	309,235,544
6Ag0002452	*AsNF-YA13*	297	894	32.55	10.22	373,120,282	373,124,392
6Cg0000792	*AsNF-YA14*	282	849	30.38	10.92	79,265,955	79,269,635
6Dg0001472	*AsNF-YA15*	286	861	31.06	10.74	273,683,010	273,686,695
7Cg0001079	*AsNF-YA16*	268	807	28.86	9.37	75,079,133	75,083,514
7Cg0001271	*AsNF-YA17*	335	1008	35.84	9.68	96,836,056	96,840,390
7Cg0002948	*AsNF-YA18*	212	639	22.78	8.41	538,061,666	538,065,932
7Cg0002989	*AsNF-YA19*	340	1023	36.84	9.03	542,492,173	542,496,516
1Ag0001462	*AsNF-YB01*	235	708	24.89	8.03	328,356,026	328,359,977
1Ag0002100	*AsNF-YB02*	175	528	19.52	8.86	377,567,128	377,570,424
1Ag0002988	*AsNF-YB03*	203	612	21.84	8.03	469,647,783	469,651,653
1Dg0002115	*AsNF-YB04*	127	384	13.97	9.6	361,279,521	361,283,247
2Ag0000346	*AsNF-YB05*	327	984	37.46	5.51	111,780,498	111,791,603
2Cg0001136	*AsNF-YB06*	327	984	37.51	5.32	267,329,199	267,339,388
2Dg0002913	*AsNF-YB07*	327	984	37.46	5.51	332,892,904	332,903,907
3Ag0002267	*AsNF-YB08*	168	507	18.3	9.58	422,858,420	422,860,977
6Ag0000977	*AsNF-YB09*	266	801	28.5	6.7	223,089,489	223,090,577
6Ag0002445	*AsNF-YB10*	223	672	23.17	6.02	372,493,983	372,494,996
7Cg0002053	*AsNF-YB11*	477	1434	53.97	5	228,428,924	228,434,292
1Ag0003132	*AsNF-YC01*	342	1029	38.45	8.07	484,285,698	484,291,324
2Dg0000373	*AsNF-YC02*	228	687	24.63	6.52	42,002,302	42,003,253
4Ag0000220	*AsNF-YC03*	254	765	27.97	5.48	89,431,676	89,437,376
4Cg0002331	*AsNF-YC04*	255	768	27.97	5.46	307,658,829	307,671,263
4Dg0000551	*AsNF-YC05*	237	714	25.95	4.57	122,027,701	122,033,045
5Cg0002360	*AsNF-YC06*	227	684	24.12	5.24	529,492,563	529,493,565

## Data Availability

The original contributions presented in this study are included in the article/Supplementary Materials. Further inquiries can be directed to the corresponding authors.

## Supplementary Materials

The following supporting information can be downloaded at https://www.mdpi.com/article/10.3390/plants15162466/s1, Figure S1: Phenotypes of control and *AsNF-YC02/06* VIGS-knockdown plants. Table S1: The coding sequences of *AsNF-Ys*; Table S2: The protein sequences of AsNF-Ys; Table S3: The accession numbers of genes used in the phylogenetic tree; Table S4: Newick file of the phylogenetic tree; Table S5: Motif sequence; Table S6: Classification of duplication types of *AsNF-Y* genes in allohexaploid oat; Table S7: Ka/Ks analysis of duplicated *AsNF-Y* gene pairs; Table S8: The promoter sequences of *AsNF-Y*; Table S9: List of *cis*-regulatory elements in the promoters of *AsNF-Y* genes; Table S10: The expression level of *AsNF-Y* under drought stress; Table S11: The expression level of *AsNF-Y* under high temperature stress; Table S12: The expression level of *AsNF-Y* under salt stress; Table S13: The expression level of *AsNF-Y* under alkali stress; Table S14: The VIGS fragments of *AsNF-YC02* and *AsNF-YC06*; Table S15: All primers used in this study.
