# Supplementary material for: Genome-Wide Characterization of Nuclear Factor Y (NF-Y) Transcription Factors in Allohexaploid Oat
Source: Plants (Basel). 2026 Aug 14;15(16):2466. doi: 10.3390/plants15162466 (PMC13516234; doi:10.3390/plants15162466)
Supplement: Supplementary file 1 [file plants-15-02466-s001.zip › Supplementary Figure.pdf]

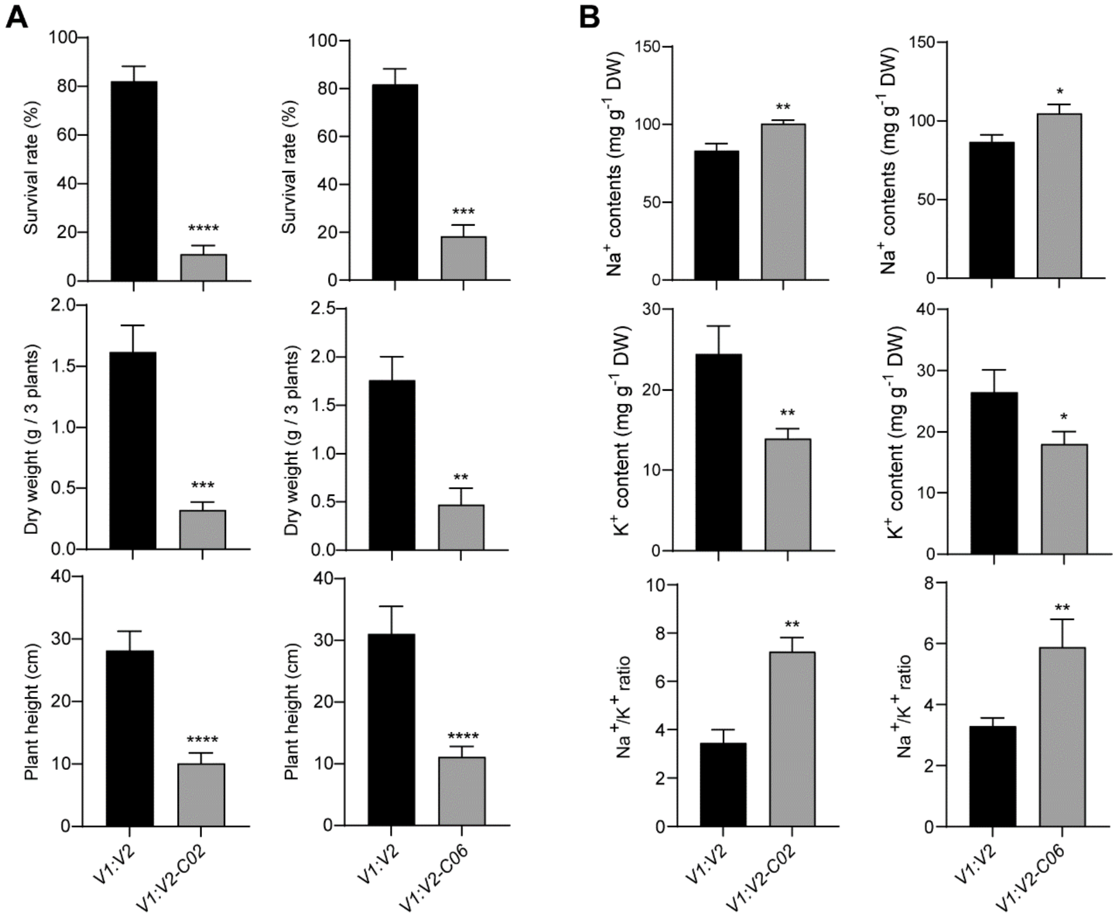


**Supplemental Figure S1.** Phenotypes of control and *AsNF-YC02/06* VIGS-knockdown plants. (A) The survival rate, dry weight and plant height of control (*V1:V2*) and VIGS-knockdown groups (*V1:V2-C02* and *V1:V2-C06*). (B) The $Na^+$ content, $K^+$ content and $Na^+/K^+$ ratio of control (*V1:V2*) and VIGS-knockdown groups (*V1:V2-C02* and *V1:V2-C06*). Values are means ± SD from two independent experiments. The asterisks indicate significance, as determined by a Student's t-test (**P* < 0.05, ***P* < 0.01; ****P* < 0.001, *****P* < 0.0001 ).
